# Temporal trends, sex differences, and age-related disease influence in Neutrophil, Lymphocyte count and Neutrophil to Lymphocyte-ratio: results from InCHIANTI follow-up study

**DOI:** 10.1186/s12979-023-00370-8

**Published:** 2023-09-04

**Authors:** Raffaello Pellegrino, Roberto Paganelli, Angelo Di Iorio, Stefania Bandinelli, Antimo Moretti, Giovanni Iolascon, Eleonora Sparvieri, Domiziano Tarantino, Luigi Ferrucci

**Affiliations:** 1 Department of Scientific Research, Off-Campus Semmelweis University, Campus Ludes, 6912 Lugano-Pazzallo, Switzerland; 2 Saint Camillus International University of Health and Medical Sciences, Rome, Italy; 3 Department of Innovative Technologies in Medicine & Dentistry, University “G. d’Annunzio”, Viale Abruzzo 322, 66100 Chieti-Pescara, Italy; 4 Geriatric Unit, Azienda Toscana Centro, Florence, Italy; 5 Department of Medical and Surgical Specialties and Dentistry, University of Campania “Luigi Vanvitelli”, 80138 Naples, Italy; 6 Department of Internal Medicine, ASL Teramo, Teramo, Italy; 7 Department of Public Health, University of Naples Federico II, 80131 Naples, Italy; 8 Longitudinal Studies Section, Translational Gerontology Branch, National Institute on Aging, National Institutes of Health, 21224 Baltimore, MD USA

**Keywords:** Neutrophils, Lymphocytes, Neutrophils-to-Lymphocytes-ratio, Observational study, Longitudinal study, Temporal-trends, Aging, Gender-dimorphism

## Abstract

**Background:**

Neutrophils and lymphocytes represent the larger percentage of all white blood cells, they vary with age, with a progressive increase of the ratio in the first years of life, and then tend to remain at similar levels in steady state condition during adult age. Neutrophils to lymphocytes-ratio (NL-ratio) was proposed as an effective and low-cost marker to monitor and predict the evolution of several clinical conditions. The main objective of the study is to analyze its temporal trend variation, over twenty years’ follow-up, according to age, sex, and main clinical diagnosis, in a large representative Italian population.

**Methods:**

The InCHIANTI study enrolled representative samples from the registry list of two towns in Tuscany, Italy. Baseline data were collected in 1998, and last follow-up visits were made in 2015-18. 1343 out of the 1453 participants enrolled were included, and consented to donate a blood sample. All subjects were assessed and followed for life-style, clinical condition, physical performance, and underwent an instrumental diagnostic session.

**Results:**

The NL-ratio showed a statistically significant interaction between birth-cohort and time of the study (p-value = 0.005). A gender dimorphism was recognized in the neutrophils absolute count and in the NL-ratio. Moreover, in female participants only, those who reported CHF had lower neutrophil-count and NL-ratio; whereas an increase in creatinine clearance was directly associated with NL-ratio. In male subjects, an increase of BMI was inversely associated with both NL-ratio and neutrophils-count during the follow-up; a similar association but in the opposite direction was observed in female participants.

**Conclusion:**

NL-ratio is a more reliable predictor of healthy aging than absolute lymphocytes and/or neutrophils counts. It is associated with the changes induced by disease, lifestyle, and environmental challenges in the immune system. NL-ratio confirms the gender dimorphism in the occurrence of inflammation-driven diseases, thus providing additional evidence for the necessity of tailored sex-specific measures to prevent and treat such diseases.

**Supplementary Information:**

The online version contains supplementary material available at 10.1186/s12979-023-00370-8.

## Introduction

The immune system is a homeostatic system that contributes to the appropriate function of the whole organism [1]. With aging, a less efficient immune system predisposes subjects to an increased risk of age-related morbidity and mortality [2]. Immunosenescence is explained by an imbalance between inflammatory and anti-inflammatory networks, resulting in the low-grade chronic pro-inflammatory status which has been termed inflammaging [3]. Underlying inflammatory processes have been recognized to be co-involved in the genesis and in the perpetuation of different ailments such as ischemic heart disease, stroke, cancer, diabetes mellitus, neurodegenerative conditions, osteoporosis and sarcopenia [4].

Immunosenescence is characterized by high inter-individual heterogeneity in adaptive and in innate immunity responses [5], though the efficacy of an immune response is also affected by changes in absolute numbers (or in the relative proportion) of immune cell subpopulations [1]. An increase in the frequency of an immune cell lineage does not necessarily reflect a good or bad response to a stressor, but according to the remodeling theory of aging, it should be considered the result of a successful or unsuccessful adaptation [6].

The recent COVID-19 pandemic has sparked a revived interest in the variation of cell counts such as neutrophils, lymphocytes, or their ratio, considered as possible markers of disease outcome, and not only in the elderly [7]. Consequently, a boosted circulating innate (neutrophilia) and depressed circulating adaptive immunity (lymphopenia) have been associated with worse disease outcomes and/or severe organ damage in different settings [8].

In more general terms, Wilson et al demonstrated a reduced neutrophil phagocytosis, with reduced trap formation, inaccurate migration and failure to prevent apoptosis, during aging [9]. The same group demonstrated that frailty, an augmented susceptibility to stress damage in the elderly, was associated with neutrophil chemotaxis defect in aged, compared to young not frail subjects [9].

T cells represent the overwhelming majority of circulating lymphocytes, with relative increase (despite absolute decrease of lymphocyte counts) with age [10]. With advancing age, naive T cells are gradually being replaced by highly differentiated memory and senescent cell types. Senescent T cells are dysfunctional immune cells, without division capacity, resistant to apoptosis, and secrete large amounts of pro-inflammatory mediators [11].

The neutrophil-to-lymphocyte ratio (NL-ratio) is a composite marker of the absolute peripheral count of both types of cells, and it has been used to indicate the immune-inflammatory activities of neutrophils and lymphocytes in several clinical conditions such as cancer progression [12], cardiovascular diseases [13], kidney diseases [14], and hypertension [15]. In the Rotterdam study, a long-standing population-based prospective cohort study on aging, NL-ratio levels were independently associated with an increased risk of cardiovascular and all-cause mortality, but not with cancer mortality [16].

No data reporting temporal trends in the variation of absolute counts of lymphocytes and neutrophils, and in their ratio, are available in an aging population, also accounting for gender differences, and in relation to the main clinical conditions. Therefore, the main objective of this study is to assess factors that affect age-trajectories of circulating neutrophils and lymphocytes in the InCHIANTI study, a large longitudinal study conducted in a representative population of the Italian population.

## Results

The main characteristics of the population enrolled in the study, both at baseline and at subsequent follow-up times are reported in Table 1. The absolute number of deaths was reported as the number of events registered between consecutive times of the study, and as a percentage of the entire sample (n = 1453). Events reported in the last column (Follow-up 4), refer to deaths that occurred after the last follow-up (from 2015 to 2018). In a period of 20 years 851 deaths were registered (58.6% of the entire sample).

**Table 1:** Descriptive of the study population according to time of follow-up

	Baseline	Follow-up 1	Follow-up 2	Follow-up 3	Follow-up 4
Number of subjects	1343	1038	948	834	469
Age at baseline (yy)	68.86±15.63	67.18±15.43	65.63±15.45	64.37±14.94	59.53±15.81
Age chronological (yy)	68.86±15.63	70.23±15.44	71.71±15.46	73.52±14.97	74.00±15.83
Sex female n (%)	745 (55.47)	574 (55.30)	532 (56.12)	467 (56.0)	258 (55.01)
Death n (%) §	144 (9.91)	155 (10.66)	140 (9.63)	215 (14.79)	197 (13.56)
Cigarettes smoking habit n (%)	240 (17.87)	155 (14.93)	118 (12.45)	87 (10.43)	43 (9.17)
Neutrophils count (K/µL)	3.75±1.26	3.60±1.16	3.67±1.25	3.73±1.41	3.56±1.35
Lymphocytes count (K/µL)	1.91±0.65	1.93±0.65	1.94±0.65	1.95±0.66	1.94±0.62
Neutrophil to Lymphocyte ratio (K/µL)	2.17±1.00	2.01±0.88	2.19±1.18	2.20±1.28	2.07±1.41
Log NLR* (K/µL)	0.68±0.42	0.62±0.41	0.68±0.43	0.67±0.47	0.60±0.48

* Log NLR = logarithm of Neutrophil to Lymphocyte ratio;

§ Deaths were reported as the number of events registered between time of the study, and as percentage of the entire sample (n = 1453), those events reported in column Follow-up 4 refer to events registered after the last contact from 2015 to 2018.

Chronological age represents the population’s mean age at the specific follow-up; whereas, age at baseline represents the mean age of those subjects who were alive, in the specific follow-up, at the enrollment in the study. The mean leukocytes count, and their ratio showed small variation across the study follow-up.

### Age and time effect

**Fig. 1:**
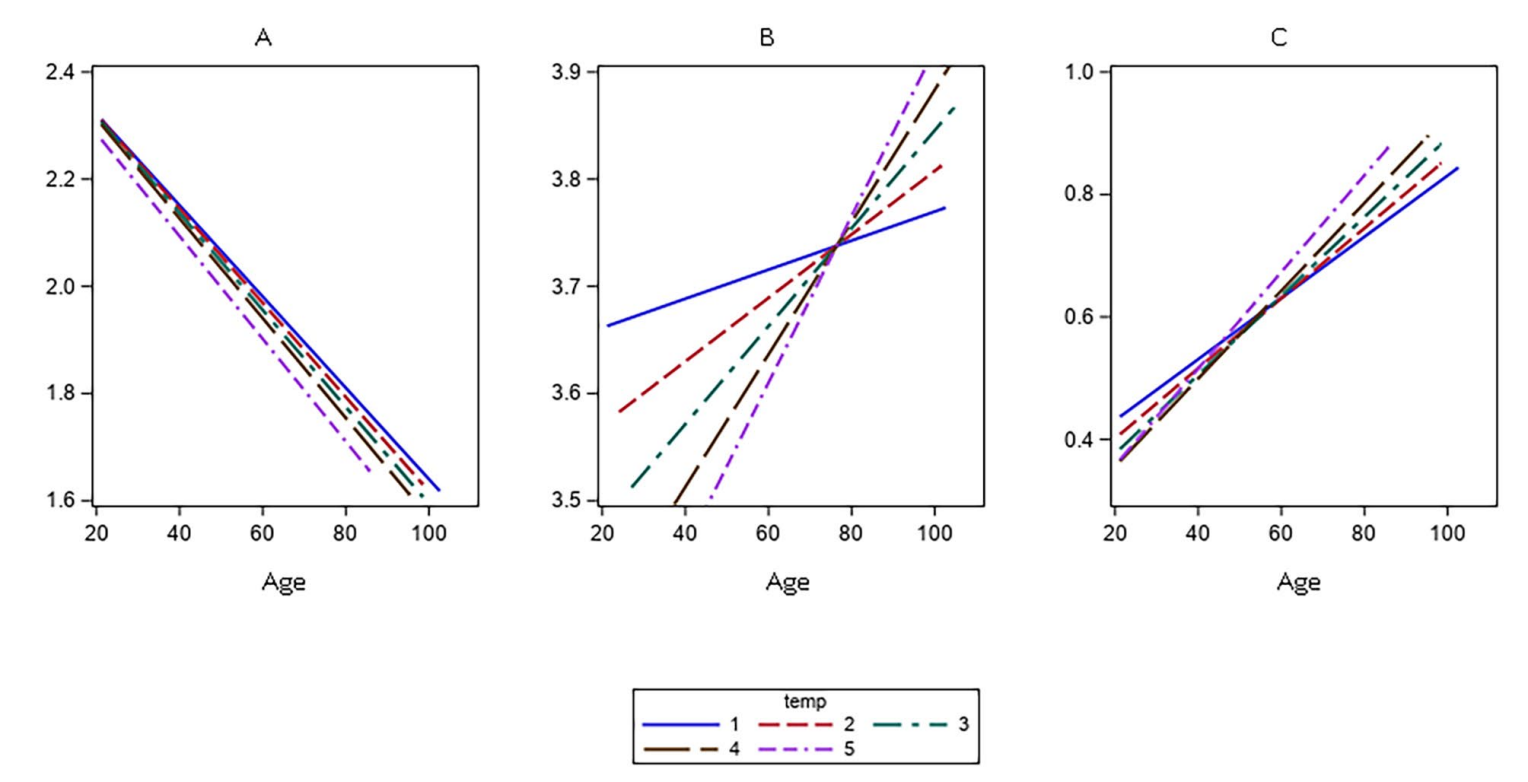
Linear Mixed Model, slopes for the associations between lymphocytes-count (**A**), neutrophil-count (**B**), and neutrophil to lymphocytes ratio logarithm (**C**), and chronological-age according to times of the study

Lymphocyte counts decreased linearly according to year of birth-cohort (p < 0.001), and no significant differences were found across the follow-up times (Fig. 1.A). On the contrary, neutrophil counts had a direct association with birth-cohort, but different slopes for different times of the study were found (Fig. 1B; for the interaction p-value = 0.04). Also, the NL-ratio showed a linear direct association with birth cohort, and the slopes of this association varied at different follow-up times (Fig. 1C; for the interaction p-value = 0.005).

### Sex effect

We observed no gender dimorphism for lymphocyte counts (Table 2 section-A); whereas for neutrophil counts and the NL-ratio different slopes were found for the association of sex with birth-cohort, but no differences for the interaction between the follow-up times of the study and sex (Table 2 sections B-C). Interestingly, the NL-ratio shows a multiplicative effect for age and time of the study (0.0008 ± 0.0003; p-value = 0.002).

**Table 2:** Linear mixed models, analyzing variation of lymphocytes, neutrophils, and neutrophils to lymphocytes-ratio, according to age, sex, and across study follow-up.

			Lymphocytes *coefficient (SE)*	p-value	Neutrophils *coefficient (SE)*	p-value	Log-NL-ratio *coefficient (SE)*	p-value
Initial status	Intercept	γ _00_	2.634 (0.245)	< 0.001	2.703 (0.459)	< 0.001	0.021 (0.153)	0.89
	Age	γ _01_	-0.009 (0.003)	0.02	0.017 (0.007)	0.008	0.009 (0.002)	< 0.001
	Sex	γ _02_	-0.107 (0.148)	0.47	0.613 (0.275)	0.03	0.215 (0.092)	0.02
Rate of change	Intercept	γ _10_	-0.008 (0.031)	0.80	-0.089 (0.063)	0.16	-0.027 (0.020)	0.19
	Interaction Time*Age	γ _11_	-0.0006 (0.0004)	0.14	0.0014 (0.0008)	0.08	0.0008 (0.0003)	0.002
	Interaction Time*Sex	γ _12_	0.018 (0.012)	0.15	0.003 (0.025)	0.90	-0.009 (0.008)	0.28
	Interaction Age*Sex	γ _13_	0.001 (0.002)	0.87	-0.011 (0.004)	0.006	-0.003 (0.001)	0.02
Level 1	Within person	δ ^2^_ε_	0.107 (0.003)	< 0.001	0.678 (0.021)	< 0.001	0.069 (0.002)	< 0.001
Level 2	In initial status	δ ^2^_0_	0.354 (0.021)	< 0.001	1.036 (0.086)	< 0.001	0.113 (0.009)	< 0.001
	In rate of change	δ ^2^_1_	0.020 (0.002)	< 0.001	0.053 (0.008)	< 0.001	0.006 (0.001)	< 0.001
	Covariance	δ _01_	-0.034 (0.005)	< 0.001	-0.089 (0.023)	< 0.001	-0.009 (0.002)	< 0.001
		AIC	6627		13824		3540	
		BIC	6685		13882		3598	

Note Abbreviations: γ_00_ = intercept of the average trajectory; γ_01_ = intercept of the age trajectory; γ_02_ = intercept of the sex trajectory; γ_10_ = intercept time effect of the trajectory for the time of the study effect; γ_11_ = slope of the trajectory for the interaction between time and age; γ_12_ = slope of the trajectory for the interaction between time and sex; γ13 = slope of the trajectory for the interaction between age and sex; δ^2^_e_ = within-person variance components; δ^2^_0_ = in initial status variance components; δ^2^_1_ = in rate of change variance components; δ_01_ = covariance estimate; AIC = Akaike information criterion; BIC = Bayesian information criterion.

### Diseases effect

Lymphocyte counts were not associated with any of the diseases considered, namely: diabetes, stroke, cancer, congestive heart failure, and renal failure (data not reported). Due to gender dimorphism in the neutrophil counts and NL-ratio, subsequent analyses were conducted separately in males and females. In both sexes, not statistically significant first and second-order effect for neoplastic diseases, diabetes, and stroke could be found in the models with NL-ratio and neutrophil count variations as dependent variables (additional Tables 2–4).

**Fig. 2:**
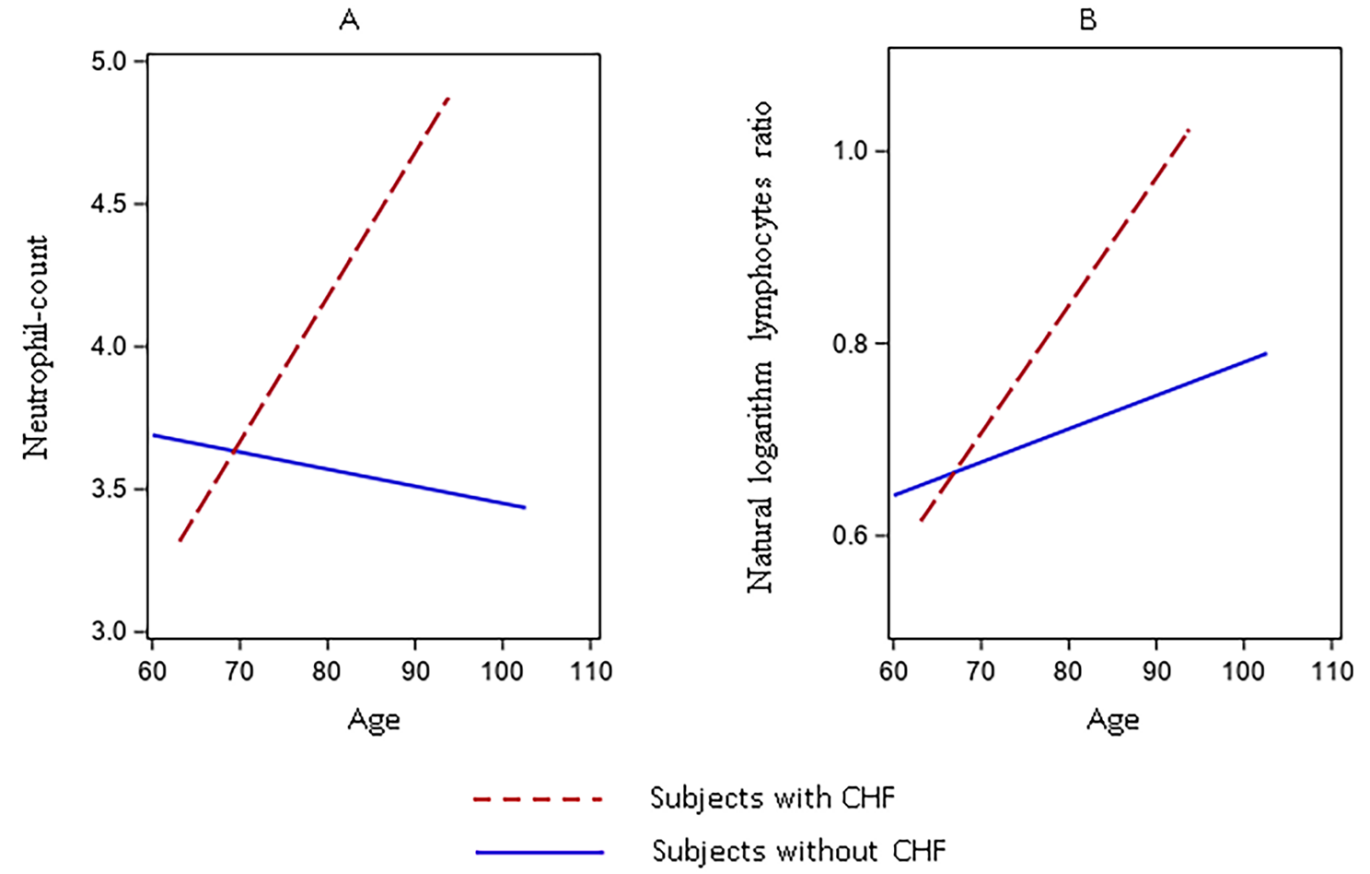
Linear Mixed Model, slopes for the associations between neutrophil-count (**A**), and neutrophil to lymphocytes ratio natural logarithm (**B**) and age in female subjects with a CHF diagnosis

In females only, congestive heart failure (CHF) was directly associated with the NL-ratio and neutrophil count (0.782 ± 0.403; p = 0.05, and 39.698 ± 12.068; p = 0.001, respectively), and for both markers the second order (age for CHF interaction) effect resulted in an attenuation of the associations (-0.011 ± 0.005; p = 0.03, and − 0.057 ± 0.015; p < 0.001, for NL-ratio and neutrophil counts, respectively) (Figs. 2.A and B and additional Table 5). A similar picture was observed for Creatinine Clearance (CC), which was directly associated with the NL-ratio and neutrophil count (0.004 ± 0.001; p = 0.005; 0.019 ± 0.005; p < 0.001, respectively), and for both, the second order (age for CC interaction) effect resulted in an attenuation of the associations (-0.0001 ± 0.00002; p-value < 0.001; -0.0003 ± 0.0001, p-value < 0.001, NL-ratio and neutrophil count respectively) (additional Table 6),

### Other potential confounders

In male subjects, the interaction between the times of the study and BMI was inversely associated with both the NL-ratio and the neutrophil count (-0.006 ± 0.002; p < 0.001; -0.016 ± 0.005; p = 0.003, respectively). The opposite was observed for females, where a direct association was found (0.002 ± 0.001, p = 0.04; 0.007 ± 0.003, p = 0.03) (additional Table 7).

Finally, cigarette smoking and alcohol intake were not associated with any of the parameters evaluated (lymphocytes count, neutrophil count, and NL-ratio).

## Discussion

The main findings of this study demonstrate that only the NL-ratio has a direct association with aging, which is demonstrated by a the statistically significant interaction between the birth-cohort effect and time effect. A gender dimorphism was found for the neutrophil absolute count and NL-ratio; for the same birth year, females had a lower number of neutrophils and a lower NL-ratio, compared to males.

Lymphocytes absolute number decreases inversely to birth-cohort, independently from sex. Sex modulates these two blood markers when categorized according to CHF and Creatinine Clearance. An inverse correlation between the birth-cohort and both neutrophils count, and NL-ratio was present only in females who developed CHF. In this same female group, higher values of all markers were measured in younger subjects, having similar CC values. Body mass index modulates the association between the blood markers and age differently according to sex, inversely in males and directly in females.

### Aging

Circulating blood cells such as leukocytes, lymphocytes, and neutrophils, are widely utilized as markers of aging-related systemic inflammation. One of the most economical and widely available clinical markers of peripheral inflammation is the NL-ratio. Variation in the NL-ratio has previously been reported to be predictive of poorer prognosis [17], longer stay after hospitalization for major illnesses, frailty [18] and disability. Interestingly NL-ratio was also reported to be cross-sectionally associated with Alzheimer’s Dementia (AD) [19]; but in the Australian Imaging, Biomarker & Lifestyle Flagship Study of Ageing (AIBL cohort), longitudinal analysis of NL-ratio variation across time was limited for the problem of diagnosing AD, and unable to predict the transition from mild cognitive impairment to AD [20]. Such correlations were weak, and disappeared when age and sex were considered, indicating that those covariates, rather than the underlying disease process, drove the changes observed in the ratio [20]. In the Rotterdam study the NL-ratio levels were associated with an increased risk of all-cause mortality, independently from age; however the authors did not assess a potential association between age and NL-ratio [16]. In the InCHIANTI study, the NL-ratio showed a departure from a linear trend, but maintained a direct correlation with age, and, more interestingly, with the interaction between birth-cohort and times of the study (aging effect). This would possibly suggest that the NL-ratio might be a worthwhile and stable marker of healthy aging.

Both the innate and the adaptive immune systems are dynamically remodeled with aging, in a process which is known as immunosenescence, characterized by great heterogeneity [21]. Adaptive immunity changes can be tracked to a reduction of lymphocytes, mainly due to a decreased thymic output [22], and with an imbalanced CD4:CD8 ratio, a decrease in the number of CD4 cells and a contextual increase in the number of CD8 cells [23].

Peripheral T lymphocytes encompass a heterogeneous mix of cellular types, namely naive, effector, and memory cells. They have different lifespan and continuously recirculate through the lymphatic system. Investigations into the dynamics of T cell turnover have demonstrated that most peripheral T cells can remain in a quiescent state for prolonged periods, spanning months in rodents and even years in humans. Notably, typical naive T cells are known for their remarkable longevity as they persist as quiescent cells [24]. Conversely, effector and memory T cells exhibit a more rapid turnover, indicating their active involvement in immune responses [25]. Senescent-like features in highly differentiate T cells are characterized by a reduced proliferation rate, shorter telomere length and increased levels of p38 [26]. These senescent-like T cells express NK cell receptors and high levels of cytotoxic molecules, that might be responsible for tissue damage [27]. Also B cells show senescence signature mainly characterized by a pro-inflammatory senescence associated secretory phenotype [28]. An expanded subset of B cells that accumulate with age (age-associated B cells) [29] plays a significant role in various aspects of immunosenescence [30] and they are found at higher levels in autoimmune and autoinflammatory diseases [29]. The numbers of B lymphocytes are greatly reduced in aged individuals [10] but their function is important for the aging process.

Neutrophils are part of the innate immune system and represent the main effector cells against bacterial infections. Neutrophils also play a critical role in disease control, as in cancer [31]. The absolute number of neutrophils does not change significantly in immunosenescence [9]; they show a short life [32] (less than one day), coupled with a high production rate in the bone marrow (5–10×10^10^ neutrophils-day) [33]. During aging neutrophils show a decline in phagocytic functions that lead to an imbalance of tissue homeostasis [34]. Their senescence features include shortened telomere length, reduced proliferation, and a pro-inflammatory senescence associate secretory phenotype [34, 35].

In our study, the absolute number of circulating lymphocytes decreased with age independently from sex, whereas neutrophil count increased. This accounts for the declining NL ratio, however the reasons for the opposite trend are uncertain.

These results could be explained considering that immune system function undergoes a profound remodeling with age, even if it was extremely interindividual heterogeneous (immunobiography) [36]. Elderly subjects show an increased risk for infective and degenerative disease manifestations, and that is thought to be a phenomenon associated with a less efficient adaptive immune response [4]. On the contrary, some features of innate immunity seem to be preserved in immunosenescence; for example, inflammation is not dampened with age [37]. The aging-related exhaustion of the adaptive immune system recognized large and complex interactions of factors as genomic instability (shortening of telomeres) [38], epigenetic regulation (DNA methylation, histone modification, and noncoding RNAs) [39], damage to mitochondrial function (reducing energy availability), as well as hormones imbalance, multimorbidity and environmental causes[40, 41]. The prospective character of the study, with 20 years of follow-up (from 1998 to 2018), has enabled us to better capture this trend in the aging population.

### Gender

Sex differences in the immune responses have been extensively demonstrated [40, 42]. Generally, females have a more efficient innate and adaptive immune response than males, and this apparent beneficial effect, is counterbalanced by female increased susceptibility to inflammatory and autoimmune diseases [43]. Reduced immune function in men might represent an example of pleiotropic antagonism, i.e. a side effect of positive selection for other traits, such as reproductive success or enhanced metabolism [44].

Lymphocyte absolute numbers did not differ between sexes, but a gender dimorphism was found in neutrophils count, with females showing higher numbers and NL-ratio, compared to males. With aging this sex-biased gap tended to be tapering, as demonstrated by the significant inverse interaction between age and sex [45]. These differences might plausibly be due to age-related changes in hormonal levels and immunological remodeling. For example, after a single i.m. injection of 17beta-oestradiol in male subjects, the number of neutrophils in blood doubled, although no significant changes in adhesion molecules were measured [46]. Androgens suppress the pro-inflammatory responses via inhibition of leukotriene formation in neutrophils [47]; progesterone enhance prostaglandin production of activated macrophages in female murine models inhibiting nitric-oxide production [48], whereas in a male murine model, estrogen treatment improves cellular immunity through NF-kB activation and reduced IL-6 production [49].

An alternative hypothesis explaining the gender differences considers the sexually dimorphic gene expression. The X chromosome is more complex than the Y chromosome, and contains many genes involved in immune function [50], which are thought to be partly responsible for the hyperresponsiveness of the female immune system; for example, males experience more frequent severe infections, on the contrary females are more prone to autoimmune diseases [51]. Those differences are attributable to X chromosome inactivation (XCI) during embryogenesis, female-specific mechanism to equalize gene expression between the sexes [45], and the genes escape silencing, a possible mechanism that would account for the overexpression of X-linked immune genes [52]. Approximately a quarter of X-linked genes are estimated to constitutively escape from XCI in humans and may contribute to the female autoimmune predisposition [52], with higher serum IgM, higher number of B cells, and higher percentage of CD4 + T-helper cells in females compared to males [52].

### Diseases

Myocardial infarction is the leading cause of the development of CHF. Neutrophils are the first cells recruited to the site of myocardial injury, and they are of paramount importance in the clearance of necrotic tissue, for the local resolution of inflammation, and in tissue homeostasis [53]. To explain the reason for the increase in the neutrophil count and the NL-ratio only in female subjects with CHF, we must consider reasons beyond the immunological gender dimorphism. Sex differences are reported in heart tissue from the early embryogenesis, and they are epigenetically perpetuated. Those differences may have sex-specific repercussions during organogenesis, persisting through adulthood and producing a different response of tissues to injury [54].

Chronic kidney disease (CKD) progression has been associated with chronic inflammation and specifically with higher NL-ratio [55]. A recent meta-analysis confirmed the NL-ratio predictive value for death in CKD patients, for all causes and for specific cardiovascular mortality [55], suggesting also its use for monitoring inflammation, and renal insufficiency progression [56]. In the InCHIANTI data, creatinine clearance was directly associated with neutrophil count and NL-ratio, although in female subjects only. Younger subjects with low creatinine clearance showed higher levels of the two blood markers, and this figure is in agreement with previous findings in CKD [57].

### Body composition

Lastly, in our study an increase in BMI across the follow-up times accompanies an increase of neutrophils count and NL-ratio in females, whereas in males for an increase in BMI a decrease in both parameters was found. Differences in body composition between sexes are well documented in aging; usually, women have a higher fat mass, and men have a higher muscle mass [58], and excessive fat deposition can cause a proinflammatory state due to adipose tissue secretion of cytokines, and disturbances in metabolic homeostasis [28]. Therefore, also sexual dimorphism in lipid metabolism must be considered and could explain the link among adipose tissue, aging, and chronic inflammation. Alternatively, starvation, malnutrition, as well as single nutrient deficiencies, may modify the balance between adaptive and innate immunity, probably as a consequence of bone marrow failure [59]. Moreover, intermitting fasting and/or caloric restriction response is gender-specific [60]. Even if, in females mice caloric restriction increases adipocytes infiltration, at least in the bone marrow [61] and in the liver [62], compared to males; those observations highlighted how different energy homeostasis is governed by sex hormones, and modulates chronic low grade inflammation.

## Conclusion

In the InCHIANTI study, NL-ratio is a more reliable marker of healthy aging compared to absolute lymphocyte and neutrophil counts. Our results confirm also that immunosenescence shows a gender dimorphism, with an imbalance between adaptive and innate immunity. Disease manifestations such as age-related renal insufficiency and congestive heart failure influence immunosenescence in a gender-specific way. Lastly also lifestyle and more specifically body composition affect Inflammaging in a gender-specific way. Therefore, this study provides further evidence for the necessity of a tailored, sex-specific approach to prevention and therapy of age-related conditions.

## Method

The InCHIANTI study protocol has been described in detail elsewhere [63]. Briefly, the study was designed by the Laboratory of Clinical Epidemiology of the Italian National Institute of Research and Care on Aging (INRCA, Florence, Italy), and was performed in two small towns in Tuscany. The baseline data were collected in 1998–2000; the three-year follow-up took place in 2001–2003, the six-year follow-up in 2004–2006, the nine-year follow-up in 2007–2009, the twelve-year follow-up 2013–2014, the last follow-up was conducted between 2015 and 2018.

### Samples

1453 participants were enrolled at baseline in the InCHIANTI Study; a total of 4632 evaluations were considered (additional Table 1) for those subjects who had at least one assessment, and all variables of interest were available. Participants were all European subjects of Caucasian origin. The Ethical Committee of the Local Health Authority of Florence, Tuscany Region, approved the study protocol, and written informed consent was obtained from each participant.

### Blood cell count analysis

Assessments of the number of red blood cells, white blood cells, platelets, hemoglobin concentration and hematometric values were performed through an automated system at the Laboratory of Clinical Chemistry and Microbiological Assays, SS. Annunziata Hospital, Azienda Sanitaria 10, Florence, Italy, using a Hematology SE 9000 Autoanalyzer (Sysmex, Kobe, Japan, provided by DASIT, Milano, Italy) for the Baseline and Follow-up 1 surveys, a Coulter LH 750 Hematology Autoanalyzer (Beckman Coulter Inc, Brea, CA, USA) for the Follow-up 2 and Follow-up 3 surveys, and a Sysmex XE 2100 (DASIT – Milano) for Follow-up 4..

### Laboratory tests

Serum Creatinine Level (mg/dL) was measured by the Laboratory of Clinical Chemistry and Microbiological Assays, SS. Annunziata Hospital, Azienda Sanitaria 10, Florence, Italy, using a colorimetric assay (TP, Roche Diagnostics, GmbH, Mannheim, Germany) and a Roche analyzer (Roche Diagnostics, GmbH, Mannheim, Germany). At Baseline, the analyzer was a Hitachi 917. For the follow-ups it was a Modular P800 Hitachi. Glomerular filtration rate was calculated according to Cockcroft-Gault formula [64].

### Statistical analysis

Descriptive data are shown as mean ± standard error, and as absolute number and percentages, for continuous and categorical variables respectively. The NL-ratio, due to the non-normal distribution, was log-transformed before the primary analysis. Linear mixed models with random intercept and random slope were applied using time since baseline as the time scale. We present four models that sequentially analyze the variation of blood cell count and their ratio: the first model considers year of birth-cohort, and possible second-order interaction between birth-cohort and time of the study; the second model considers sex effect and possible second-order interactions between sex and birth-cohort, and between sex and time of the study; the third model was sex-specific and considers as dependent variables in different models the effects of diseases (oncological diseases, diabetes, stroke, congestive heart failure, and renal insufficiency using of creatinine clearance); the last model, always stratified for sex considers the variation of BMI during the follow-up times. SAS version 9.4 for Windows (SAS Institute, Inc., Cary, NC) was used for all data processing and statistical analyses. We set the level of statistical significance at p < 0.05 (2-sided).

### Electronic Supplementary Material

Below is the link to the electronic supplementary material

Supplementary Material 1

## Data Availability

The datasets used and/or analyzed during the current study are available from the responsible authors for the InCHIANTI study (Luigi Ferrucci) on reasonable request. Data of the InCHIANTI study is available to all researchers upon justified request using the proposal form available on the InChianti website (https://www.nia.nih.gov/inchianti-study, accessed on 04/13/2023).
