# Supplementary material for: Temporal trends, sex differences, and age-related disease influence in Neutrophil, Lymphocyte count and Neutrophil to Lymphocyte-ratio: results from InCHIANTI follow-up study
Source: Immun Ageing. 2023 Sep 4;20:46. doi: 10.1186/s12979-023-00370-8 (PMC10476368; doi:10.1186/s12979-023-00370-8)
Supplement: Supplementary file 1 — Supplementary Material 1 [file 12979_2023_370_MOESM1_ESM.docx]

Additional Table 1: population enrolled in the study according to follow-up and to status of contact

|  | Baseline | FU-1 | FU-2 | FU-3 | FU-4 |  |
| --- | --- | --- | --- | --- | --- | --- |
|  | 1998-2000 | 2002-2003 | 2005-2006 | 2008-2009 | 2013-2014 | 2015-2018 |
|  | 1453 | 1453 | 1309 | 1154 | 1014 |  |
| Refused |  | 127 | 65 | 83 | 63 |  |
| Not located |  | 4 | 0 | 0 | 0 |  |
| Emigrated |  | 11 | 22 | 31 | 49 |  |
| Death registered before follow-up |  | 144 | 155 | 140 | 215 | 197 |
|  |  |  |  |  |  |  |
| Home interviews | 1453 | 1167 | 1067 | 900 | 687 |  |
|  |  |  |  |  |  |  |
| Missing data, or proxy interview only, or refused to participate in blood donation. | 100 | 129 | 119 | 66 | 218 |  |
|  |  |  |  |  |  |  |
| Sample included in this study | 1343 | 1038 | 948 | 834 | 469 |  |

Additional Table 2: Linear mixed models, stratifying for sex neutrophils-count and Log-NL-ratio variation according to: time, age, and oncological diseases.

|  |  | Log-NL-ratio | | | | | |  | Neutrophils | | | | | |
| --- | --- | --- | --- | --- | --- | --- | --- | --- | --- | --- | --- | --- | --- | --- |
|  |  | Male | | | Female | | |  | Male | | | Female | | |
|  |  | β | SE | p | β | SE | p |  | β | SE | p | β | SE | p |
| Intercept | γ _00_ | 0.321 | 0.280 | 0.25 | 0.003 | 0.389 | 0.99 |  | 3.642 | 0.828 | <0.001 | 3.725 | 1.181 | 0.002 |
| Age | γ _01_ | 0.005 | 0.004 | 0.20 | 0.010 | 0.005 | 0.07 |  | 0.004 | 0.012 | 0.75 | -0.001 | 0.016 | 0.99 |
| Time | γ _10_ | -0.029 | 0.030 | 0.32 | -0.049 | 0.031 | 0.11 |  | -0.021 | 0.097 | 0.83 | -0.122 | 0.092 | 0.19 |
| Oncological (no) | γ _02_ | -0.103 | 0.286 | 0.72 | 0.466 | 0.391 | 0.23 |  | -0.505 | 0.838 | 0.55 | 0.324 | 11.857 | 0.79 |
| Age*time | γ _11_ | 0.0007 | 0.0004 | 0.06 | 0.0008 | 0.0004 | 0.02 |  | 0.001 | 0.001 | 0.81 | 0.002 | 0.001 | 0.05 |
| Age*oncological | γ _12_ | 0.001 | 0.004 | 0.78 | -0.007 | 0.005 | 0.19 |  | 0.006 | 0.012 | 0.64 | -0.006 | 0.016 | 0.73 |
| Time*oncological | γ _13_ | -0.001 | 0.018 | 0.96 | 0.003 | 0.019 | 0.89 |  | 0.004 | 0.060 | 0.95 | -0.012 | 0.057 | 0.83 |
|  |  |  |  |  |  |  |  |  |  |  |  |  |  |  |
| Initial status | δ ^2^_0_ | 0.105 | 0.012 | <0.001 | 0.119 | 0.013 | <0.001 |  | 1.032 | 0.128 | <0.001 | 1.036 | 0.117 | <0.001 |
| Covariance | δ _01_ | -0.004 | 0.003 | 0.240 | -0.011 | 0.003 | 0.001 |  | -0.095 | 0.036 | 0.008 | -0.085 | 0.031 | 0.006 |
| In rate change | δ ^2^_1_ | 0.005 | 0.001 | <0.001 | 0.006 | 0.001 | <0.001 |  | 0.059 | 0.013 | <0.001 | 0.049 | 0.011 | <0.001 |
| Within person | δ ^2^ _ε_ | 0.062 | 0.003 | <0.001 | 0.074 | 0.003 | <0.001 |  | 0.640 | 0.030 | <0.001 | 0.707 | 0.029 | <0.001 |
|  |  |  |  |  |  |  |  |  |  |  |  |  |  |  |
| AIC |  | 1450.6 |  |  | 2091.1 |  |  |  | 6109.2 |  |  | 7731.0 |  |  |
| AICC |  | 1450.7 |  |  | 2091.2 |  |  |  | 6109.3 |  |  | 7731.1 |  |  |
| BIC |  | 1499.7 |  |  | 2142.8 |  |  |  | 6158.3 |  |  | 7782.7 |  |  |
|  |  |  |  |  |  |  |  |  |  |  |  |  |  |  |

Additional Table 3: Linear mixed models, stratifying for sex neutrophils-count and Log-NL-ratio variation according to: time, age, and diabetes.

|  |  | Log-NL-ratio | | | | | |  | Neutrophils | | | | | |
| --- | --- | --- | --- | --- | --- | --- | --- | --- | --- | --- | --- | --- | --- | --- |
|  |  | Male | | | Female | | |  | Male | | | Female | | |
|  |  | β | SE | p | β | SE | p |  | β | SE | p | β | SE | p |
| Intercept | γ _00_ | 0.476 | 0.240 | 0.04 | 0.234 | 0.296 | 0.43 |  | 3.636 | 0.712 | <0.001 | 2.772 | 0.892 | 0.002 |
| Age | γ _01_ | 0.003 | 0.003 | 0.39 | 0.006 | 0.004 | 0.12 |  | 0.002 | 0.010 | 0.86 | 0.013 | 0.012 | 0.25 |
| Time | γ _10_ | -0.035 | 0.028 | 0.22 | -0.021 | 0.030 | 0.48 |  | 0.013 | 0.093 | 0.89 | -0.039 | 0.089 | 0.67 |
| Diabetes (no) | γ _02_ | -0.273 | 0.246 | 0.27 | 0.235 | 0.230 | 0.43 |  | -0.515 | 0.721 | 0.48 | 13.586 | 0.898 | 0.13 |
| Age*time | γ _11_ | 0.0007 | 0.0004 | 0.05 | 0.0007 | 0.0003 | 0.04 |  | 0.001 | 0.001 | 0.87 | 0.002 | 0.001 | 0.08 |
| Age* diabetes | γ _12_ | 0.004 | 0.003 | 0.27 | -0.003 | 0.004 | 0.41 |  | 0.008 | 0.009 | 0.41 | -0.021 | 0.012 | 0.08 |
| Time* diabetes | γ _13_ | 0.004 | 0.015 | 0.78 | -0.022 | 0.017 | 0.18 |  | -0.030 | 0.049 | 0.54 | -0.088 | 0.050 | 0.08 |
|  |  |  |  |  |  |  |  |  |  |  |  |  |  |  |
| Initial status | δ ^2^_0_ | 0.105 | 0.012 | <0.001 | 0.120 | 0.0130 | <0.001 |  | 1.030 | 0.128 | <0.001 | 1.022 | 0.117 | <0.001 |
| Covariance | δ _01_ | -0.004 | 0.003 | 0.25 | -0.012 | 0.003 | <0.001 |  | -0.095 | 0.036 | <0.001 | -0.088 | 0.031 | 0.005 |
| In rate change | δ ^2^_1_ | 0.005 | 0.001 | <0.001 | 0.006 | 0.001 | <0.001 |  | 0.059 | 0.013 | <0.001 | 0.049 | 0.011 | <0.001 |
| Within person | δ ^2^ _ε_ | 0.062 | 0.003 | <0.001 | 0.074 | 0.003 | <0.001 |  | 0.640 | 0.030 | <0.001 | 0.707 | 0.029 | <0.001 |
|  |  |  |  |  |  |  |  |  |  |  |  |  |  |  |
| AIC |  | 1449.7 |  |  | 2089.1 |  |  |  | 6109.0 |  |  | 7714.5 |  |  |
| AICC |  | 1449.9 |  |  | 2089.2 |  |  |  | 6109.1 |  |  | 7714.6 |  |  |
| BIC |  | 1498.8 |  |  | 2140.7 |  |  |  | 6158.1 |  |  | 7766.2 |  |  |
|  |  |  |  |  |  |  |  |  |  |  |  |  |  |  |

Additional Table 4: Linear mixed models, stratifying for sex neutrophils-count and Log-NL-ratio variation according to: time, age, and stroke.

|  |  | Log-NL-ratio | | | | | |  | Neutrophils | | | | | |
| --- | --- | --- | --- | --- | --- | --- | --- | --- | --- | --- | --- | --- | --- | --- |
|  |  | Male | | | Female | | |  | Male | | | Female | | |
|  |  | β | SE | p | β | SE | p |  | β | SE | p | β | SE | p |
| Intercept | γ _00_ | 0.723 | 0.456 | 0.11 | 0.248 | 0.415 | 0.55 |  | 3.878 | 1.353 | 0.004 | 2.658 | 1.257 | 0.04 |
| Age | γ _01_ | -0.001 | 0.006 | 0.86 | 0.006 | 0.005 | 0.23 |  | 0.0001 | 0.018 | 0.99 | 0.014 | 0.015 | 0.38 |
| Time | γ _10_ | -0.024 | 0.034 | 0.47 | -0.036 | 0.035 | 0.29 |  | -0.119 | 0.111 | 0.28 | -0.088 | 0.104 | 0.40 |
| Stroke (no) | γ _02_ | -0.525 | 0.459 | 0.25 | 0.224 | 0.416 | 0.59 |  | -0.726 | 1.359 | 0.59 | 1.446 | 1.261 | 0.25 |
| Age*time | γ _11_ | 0.0007 | 0.0004 | 0.07 | 0.0007 | 0.0003 | 0.03 |  | 0.0005 | 0.001 | 0.64 | 0.002 | 0.001 | 0.05 |
| Age* stroke | γ _12_ | 0.008 | 0.006 | 0.19 | -0.004 | 0.005 | 0.50 |  | 0.009 | 0.017 | 0.60 | -0.021 | 0.016 | 0.20 |
| Time* stroke | γ _13_ | -0.005 | 0.021 | 0.82 | -0.009 | 0.023 | 0.71 |  | 0.091 | 0.070 | 0.19 | -0.043 | 0.070 | 0.53 |
|  |  |  |  |  |  |  |  |  |  |  |  |  |  |  |
| Initial status | δ ^2^_0_ | 0.104 | 0.012 | <.0001 | 0.119 | 0.013 | <.0001 |  | 1.040 | 0.128 | <.0001 | 1.035 | 0.117 | <.0001 |
| Covariance | δ _01_ | -0.004 | 0.003 | 0.250 | -0.012 | 0.003 | 0.0009 |  | -0.098 | 0.036 | 0.007 | -0.087 | 0.031 | 0.005 |
| In rate change | δ ^2^_1_ | 0.005 | 0.001 | <.0001 | 0.006 | 0.001 | <.0001 |  | 0.059 | 0.013 | <.0001 | 0.049 | 0.011 | <.0001 |
| Within person | δ ^2^ _ε_ | 0.061 | 0.003 | <.0001 | 0.074 | 0.003 | <.0001 |  | 0.639 | 0.030 | <.0001 | 0.707 | 0.029 | <.0001 |
|  |  |  |  |  |  |  |  |  |  |  |  |  |  |  |
| AIC |  | 1447.6 |  |  | 2090.7 |  |  |  | 6107.3 |  |  | 7727.2 |  |  |
| AICC |  | 1447.7 |  |  | 2090.8 |  |  |  | 6107.5 |  |  | 7727.3 |  |  |
| BIC |  | 1496.7 |  |  | 2142.4 |  |  |  | 6156.4 |  |  | 7778.9 |  |  |
|  |  |  |  |  |  |  |  |  |  |  |  |  |  |  |

Additional Table 5: Linear mixed models, stratifying for sex neutrophils-count and Log-NL-ratio variation according to: time, age, and Congestive heart failure.

|  |  | Log-NL-ratio | | | | | |  | Neutrophils | | | | | |
| --- | --- | --- | --- | --- | --- | --- | --- | --- | --- | --- | --- | --- | --- | --- |
|  |  | Male | | | Female | | |  | Male | | | Female | | |
|  |  | β | SE | p | β | SE | p |  | β | SE | p | β | SE | p |
| Intercept | γ _00_ | 0.395 | 0.433 | 0.36 | -0.282 | 0.401 | 0.48 |  | 4.381 | 1.285 | <0.001 | 0.321 | 1.202 | 0.79 |
| Age | γ _01_ | 0.004 | 0.005 | 0.50 | 0.013 | 0.005 | 0.009 |  | -0.008 | 0.016 | 0.62 | 0.047 | 0.015 | 0.002 |
| Time | γ _10_ | 0.001 | 0.034 | 0.98 | -0.027 | 0.033 | 0.40 |  | 0.085 | 0.112 | 0.45 | -0.148 | 0.098 | 0.13 |
| CHF | γ _02_ | -0.182 | 0.436 | 0.67 | 0.782 | 0.403 | 0.05 |  | -1.281 | 1.291 | 0.32 | 3.970 | 1.207 | 0.001 |
| Age*time | γ _11_ | 0.0005 | 0.0003 | 0.12 | 0.0007 | 0.0003 | 0.03 |  | -0.001 | 0.001 | 0.91 | 0.002 | 0.001 | 0.03 |
| Age* CHF | γ _12_ | 0.003 | 0.006 | 0.62 | -0.011 | 0.005 | 0.03 |  | 0.0183 | 0.016 | 0.27 | -0.057 | 0.015 | <0.001 |
| Time* CHF | γ _13_ | -0.026 | 0.021 | 0.21 | -0.017 | 0.019 | 0.35 |  | -0.083 | 0.067 | 0.22 | 0.0001 | 0.057 | 0.99 |
|  |  |  |  |  |  |  |  |  |  |  |  |  |  |  |
| Initial status | δ ^2^_0_ | 0.105 | 0.012 | <0.001 | 0.117 | 0.013 | <0.001 |  | 1.034 | 0.128 | <0.001 | 0.965 | 0.113 | <0.001 |
| Covariance | δ _01_ | -0.004 | 0.003 | 0.24 | -0.011 | 0.003 | 0.001 |  | -0.096 | 0.036 | 0.008 | -0.078 | 0.030 | 0.01 |
| In rate change | δ ^2^_1_ | 0.005 | 0.001 | <0.001 | 0.006 | 0.001 | <0.001 |  | 0.059 | 0.013 | <0.001 | 0.049 | 0.011 | <0.001 |
| Within person | δ ^2^ _ε_ | 0.062 | 0.003 | <0.001 | 0.074 | 0.003 | <0.001 |  | 0.639 | 0.030 | <0.001 | 0.706 | 0.029 | <0.001 |
|  |  |  |  |  |  |  |  |  |  |  |  |  |  |  |
| AIC |  | 1449.0 |  |  | 2081.3 |  |  |  | 6107.0 |  |  | 7699.6 |  |  |
| AICC |  | 1449.1 |  |  | 2081.4 |  |  |  | 6107.1 |  |  | 7699.7 |  |  |
| BIC |  | 1498.1 |  |  | 2133.0 |  |  |  | 6156.1 |  |  | 7751.2 |  |  |
|  |  |  |  |  |  |  |  |  |  |  |  |  |  |  |

Additional Table 6: Linear mixed models, stratifying for sex neutrophils-count and Log-NL-ratio variation according to: time, age, and variation of Creatinine clearance estimated by Cockcroft-Gault formula during follow-up.

|  |  | Log-NL-ratio | | | | | |  | Neutrophils | | | | | |
| --- | --- | --- | --- | --- | --- | --- | --- | --- | --- | --- | --- | --- | --- | --- |
|  |  | Male | | | Female | | |  | Male | | | Female | | |
|  |  | β | SE | p | β | SE | p |  | β | SE | p | β | SE | p |
| Intercept | γ _00_ | 0.252 | 0.214 | 0.24 | 0.203 | 0.176 | 0.25 |  | 2.278 | 0.687 | 0.001 | 2.578 | 0.519 | <0.001 |
| Age | γ _01_ | 0.004 | 0.003 | 0.15 | 0.009 | 0.002 | <0.001 |  | 0.016 | 0.009 | 0.06 | 0.019 | 0.006 | 0.003 |
| Time | γ _10_ | 0.002 | 0.051 | 0.98 | -0.128 | 0.047 | 0.007 |  | 0.222 | 0.167 | 0.19 | -0.308 | 0.132 | 0.02 |
| Creatinine clearance | γ _02_ | -0.001 | 0.002 | 0.61 | 0.004 | 0.001 | 0.005 |  | 0.006 | 0.005 | 0.27 | 0.019 | 0.005 | <0.001 |
| Age*time | γ _11_ | 0.001 | 0.001 | 0.29 | 0.008 | 0.0004 | 0.008 |  | -0.002 | 0.002 | 0.39 | 0.003 | 0.001 | 0.02 |
| Age* Creatinine Cl | γ _12_ | 0.001 | 0.001 | 0.12 | -0.0001 | 0.00002 | <0.001 |  | -0.001 | 0.001 | 0.90 | -0.0003 | 0.0001 | <0.001 |
| Time* Creatinine Cl | γ _13_ | -0.001 | 0.001 | 0.36 | 0.0005 | 0.0003 | 0.07 |  | -0.001 | 0.001 | 0.09 | 0.001 | 0.001 | 0.21 |
|  |  |  |  |  |  |  |  |  |  |  |  |  |  |  |
| Initial status | δ ^2^_0_ | 0.099 | 0.012 | <0.001 | 0.120 | 0.013 | <0.001 |  | 1.050 | 0.128 | <.0001 | 1.153 | 0.115 | <0.001 |
| Covariance | δ _01_ | -0.003 | 0.003 | 0.39 | -0.011 | 0.003 | <0.001 |  | -0.100 | 0.037 | 0.006 | -0.091 | 0.028 | 0.001 |
| In rate change | δ ^2^_1_ | 0.005 | 0.001 | <0.001 | 0.006 | 0.001 | <0.001 |  | 0.061 | 0.013 | <.0001 | 0.034 | 0.009 | <0.001 |
| Within person | δ ^2^ _ε_ | 0.059 | 0.003 | <0.001 | 0.066 | 0.003 | <0.001 |  | 0.602 | 0.030 | <.0001 | 0.566 | 0.025 | <0.001 |
|  |  |  |  |  |  |  |  |  |  |  |  |  |  |  |
| AIC |  | 1279.9 |  |  | 1717.8 |  |  |  | 5658.0 |  |  | 6625.9 |  |  |
| AICC |  | 1280.0 |  |  | 1717.9 |  |  |  | 5658.1 |  |  | 6626.0 |  |  |
| BIC |  | 1327.9 |  |  | 1768.4 |  |  |  | 5706.0 |  |  | 6676.5 |  |  |
|  |  |  |  |  |  |  |  |  |  |  |  |  |  |  |

Additional Table 7: Linear mixed models, stratifying for sex neutrophils-count and Log-NL-ratio variation according to: time, age, and Body Mass Index variation during follow-up.

|  |  | Log-NL-ratio | | | | | |  | Neutrophils | | | | | |
| --- | --- | --- | --- | --- | --- | --- | --- | --- | --- | --- | --- | --- | --- | --- |
|  |  | Male | | | Female | | |  | Male | | | Female | | |
|  |  | β | SE | p | β | SE | p |  | β | SE | p | β | SE | p |
| Intercept | γ _00_ | 0.288 | 0.397 | 0.47 | 0.317 | 0.299 | 0.29 |  | 0.581 | 1.231 | 0.64 | 3.203 | 0.890 | <0.001 |
| Age | γ _01_ | -0.001 | 0.006 | 0.80 | 0.006 | 0.004 | 0.13 |  | 0.027 | 0.017 | 0.12 | 0.005 | 0.012 | 0.67 |
| Time | γ _10_ | 0.121 | 0.048 | 0.01 | -0.118 | 0.036 | <0.001 |  | 0.379 | 0.159 | 0.02 | -0.349 | 0.105 | <0.001 |
| BMI | γ _02_ | -0.001 | 0.015 | 0.93 | 0.006 | 0.012 | 0.60 |  | 0.102 | 0.047 | 0.03 | 0.036 | 0.036 | 0.31 |
| Age*time | γ _11_ | 0.0008 | 0.0004 | 0.04 | 0.0008 | 0.0004 | 0.02 |  | 0.001 | 0.001 | 0.56 | 0.002 | 0.001 | 0.04 |
| Age* BMI | γ _12_ | 0.000 | 0.000 | 0.22 | 0.000 | 0.000 | 0.39 |  | -0.001 | 0.001 | 0.25 | 0.000 | 0.000 | 0.33 |
| Time* BMI | γ _13_ | -0.006 | 0.002 | <0.001 | 0.002 | 0.001 | 0.04 |  | -0.016 | 0.005 | 0.003 | 0.007 | 0.003 | 0.03 |
|  |  |  |  |  |  |  |  |  |  |  |  |  |  |  |
| Initial status | δ ^2^_0_ | 0.101 | 0.012 | <0.001 | 0.117 | 0.013 | <0.001 |  | 1.005 | 0.127 | <0.001 | 1.120 | 0.119 | <0.001 |
| Covariance | δ _01_ | -0.003 | 0.003 | 0.37 | -0.009 | 0.003 | 0.005 |  | -0.087 | 0.036 | 0.015 | -0.087 | 0.029 | 0.003 |
| In rate change | δ ^2^_1_ | 0.004 | 0.001 | <0.001 | 0.005 | 0.001 | <0.001 |  | 0.054 | 0.013 | <0.001 | 0.034 | 0.009 | <0.001 |
| Within person | δ ^2^ _ε_ | 0.061 | 0.003 | <0.001 | 0.071 | 0.003 | <0.001 |  | 0.620 | 0.030 | <0.001 | 0.632 | 0.027 | <0.001 |
|  |  |  |  |  |  |  |  |  |  |  |  |  |  |  |
| AIC |  | 1329.6 |  |  | 1849.3 |  |  |  | 5780.9 |  |  | 6994.9 |  |  |
| AICC |  | 1329.7 |  |  | 1849.4 |  |  |  | 5781.0 |  |  | 6995.0 |  |  |
| BIC |  | 1377.6 |  |  | 1899.8 |  |  |  | 5828.8 |  |  | 7045.4 |  |  |
|  |  |  |  |  |  |  |  |  |  |  |  |  |  |  |
